# The effectiveness of daclatasvir based therapy in European patients with chronic hepatitis C and advanced liver disease

**DOI:** 10.1186/s12879-016-2106-x

**Published:** 2017-01-07

**Authors:** Jim Young, Nina Weis, Harald Hofer, William Irving, Ola Weiland, Emiliano Giostra, Juan Manuel Pascasio, Lluis Castells, Martin Prieto, Roelien Postema, Cinira Lefevre, David Evans, Heiner C. Bucher, Jose Luis Calleja

**Affiliations:** 1 Basel Institute for Clinical Epidemiology and Biostatistics, University Hospital Basel, Spitalstrasse 12, CH-4031 Basel, Switzerland; 2 Department of Infectious Diseases, Copenhagen University Hospital, Hvidovre, Denmark; 3 Department of Internal Medicine III, Division of Gastroenterology and Hepatology, Medical University of Vienna, Vienna, Austria; 4 NIHR Nottingham Digestive Diseases Biomedical Research Unit, University of Nottingham, Nottingham, UK; 5 Division of Infectious Diseases, Department of Medicine, Karolinska Institutet, Karolinska University Hospital Huddinge, Stockholm, Sweden; 6 Service de Gastroentérologie et Hépatologie, Hôpitaux universitaires de Genève, Geneva, Switzerland; 7 Hospital Universitario Virgen del Rocío, Seville, Spain; 8 Liver Unit, Internal Medicine Department, Hospital Universitari Vall Hebron, Barcelona, Spain; 9 Hepatology Unit, Hospital Universitario y Politécnico La Fe, Valencia, Spain; 10 Worldwide Health Economics and Outcomes Research, Bristol-Myers Squibb, Uxbridge, United Kingdom; 11 Worldwide Health Economics and Outcomes Research, Bristol-Myers Squibb, Rueil-Malmaison, France; 12 Liver Unit, Hospital Universitario Puerta de Hierro, Universidad Autonoma de Madrid, Madrid, Spain

**Keywords:** Daclatasvir, Direct-acting antivirals, Effectiveness, Hepatitis C, Sofosbuvir

## Abstract

**Background:**

There is limited evidence for the effectiveness of daclatasvir in patients whose hepatitis C threatens their life expectancy. The Named Patient Program in Europe included patients with advanced chronic hepatitis C, a life expectancy of less than 12 months and no other treatment options.

**Methods:**

A retrospective multi-country cohort of patients with chronic hepatitis C who received daclatasvir as part of the Named Patient Program in Austria, Denmark, Spain, Sweden, Switzerland and the United Kingdom. Treatment response was defined as a sustained virologic response (unquantifiable hepatitis C RNA) at 12 weeks post treatment. We summarised the characteristics of the patients in this cohort and estimated the rate of sustained virologic response for patients receiving daclatasvir and sofosbuvir with or without ribavirin using hierarchical Bayesian modelling.

**Results:**

The 249 patients included had a median age of 56 years; most were male (78%), hepatitis C genotype 1 (75%), treatment experienced (65%) and with decompensated cirrhosis (59%). Many had had a liver transplant before receiving daclatasvir (40%). Of the 249 patients, 242 patients received daclatasvir and sofosbuvir and either reached 12 weeks post treatment or died during (*n =* 9) or after treatment (*n =* 4) or were lost to follow up during treatment (*n =* 1). The estimated rate of sustained virologic response at 12 weeks post treatment was 87% (95% credible interval 75 to 94%) for previously treated genotype 1 patients with decompensated cirrhosis.

**Conclusions:**

Daclatasvir with sofosbuvir is an effective treatment in clinical practice for hepatitis C genotype 1 patients with decompensated cirrhosis.

**Electronic supplementary material:**

The online version of this article (doi:10.1186/s12879-016-2106-x) contains supplementary material, which is available to authorized users.

## Background

Chronic hepatitis C often leads to cirrhosis and hepatocellular carcinoma [1]. While the prevalence of hepatitis C virus (HCV) infection is expected to decline worldwide, the number of individuals with end stage liver disease is projected to rise [2] and with it, the cost of healthcare for these individuals [3]. Earlier standard treatment with subcutaneous pegylated interferon alfa and oral ribavirin achieved sustained virologic response (SVR) rates of only 50% or less in patients infected with HCV genotype 1 [4], although SVR rates were as high as 60 to 80% in genotypes 2, 3 and 6 [4, 5]. In addition, this treatment caused potentially serious side effects and was therefore contraindicated in patients with decompensated cirrhosis [4].

Daclatasvir, a HCV NS5A replication complex inhibitor, has shown encouraging results in clinical trials across multiple genotypes, both in interferon-based regimens and in all oral regimens without interferon [6, 7]. The combination of daclatasvir and sofosbuvir, a nucleotide analogue HCV NS5B polymerase inhibitor, has shown high rates of SVR in patients with HCV genotype 1 [8, 9]. However there is limited evidence for the effectiveness of this treatment combination in patients whose advanced cirrhosis threatens their life expectancy [10–12]. There is also uncertainty about the optimal duration of treatment and whether ribavirin should be added to the combination [10–12].

A Named Patient Programme (NPP) for daclatasvir in Europe included patients from 2012 to 2015 with advanced chronic HCV, a life expectancy of less than 12 months and no other treatment options. Here we summarise the characteristics of the patients in this programme and estimate their rate of SVR when given daclatasvir in combination with sofosbuvir.

## Methods

### Patients

The first use of daclatasvir in Europe outside clinical trials was via Bristol-Myers Squibb’s (BMS) early access programmes. The NPP was one of these programmes. Patients were eligible to participate in the NPP if according to their physician: (1) the patient was an adult with chronic hepatitis C (regardless of genotype) at high risk of decompensation or death within 12 months; and (2) there were no satisfactory alternative treatments options for the patient and available treatment options had been exhausted; and (3) the patient was ineligible to participate in a clinical trial where his or her infection might be treated satisfactorily or there was no ongoing clinical trial in the patient’s country of residence.

Collaborative agreements were later signed between BMS and HCV registries or healthcare institutions with HCV databases in six European countries. These agreements allowed the transfer of individual patient data for patients in a NPP to a third party, the Basel Institute for Clinical Epidemiology and Biostatistics, for analysis. BMS did not have any access to individual patient data. The institutions providing data were: the Department of Internal Medicine III, at the Medical University of Vienna (Austria); eight hospitals in the Danish Database for Hepatitis B and C (Denmark); the Hepa C registry (Spain); the Karolinska Institutet, at Karolinska University Hospital Huddinge (Sweden); the University Hospital Geneva (Switzerland); and seven hospitals in the HCV Research UK registry (United Kingdom). All HCV registries or databases used in this study contained data prospectively collected from adult patients who had given informed consent. We planned to also include data from Italy but agreements with suitable institutions have yet to be signed.

To be eligible for this retrospective cohort study, patients had to start any daclatasvir containing regimen under a NPP (and not under any other early access programme) and have data in one of the above HCV databases. Patients were recruited from December 2012 until August 2014 when daclatasvir was approved for use in Europe but the exact dates varied between countries because in some countries, the NPP was replaced by another early access program.

### Statistical methods

The primary objective of this study was to estimate the effectiveness of daclatasvir in combination with either sofosbuvir or simeprevir as measured by the rate of sustained virologic response at 12 weeks after the end of treatment (SVR12). However only a single patient received daclatasvir with simeprevir, so our analyses were restricted to patients receiving daclatasvir and sofosbuvir, with or without ribavirin. SVR12 was defined as HCV RNA either undetectable or below the assay's lower limit of quantification.

Secondary objectives were to summarise the demographic and clinical characteristics of patients receiving daclatasvir and to assess effectiveness using other measures. Other measures of effectiveness available from all registries included the virologic response at the end of treatment and the sustained virologic response at four weeks after the end of treatment (SVR4).

The protocol (ClinicalTrials.gov NCT02531269) specified an intent-to-treat analysis such that failure to achieve a SVR was assumed if a patient died or was lost to follow up, or if a response was missing and sufficient time had elapsed since the patient finished treatment for that response to be measureable. There was an appreciable rate of mortality in these patients. As a consequence, a decision was made prior to any modelling to add an as-treated analysis in which patients with no recorded response were excluded (either because of death, loss to follow up or a missing response). In both intent-to-treat and as-treated analyses, a SVR at four or 12 weeks was assumed where a response was missing but the patient had a SVR at a later date.

SVR rates were estimated using hierarchical Bayesian modelling because small samples were anticipated from most databases. Each SVR rate was estimated under four models of increasing complexity. In all models we assumed responses were binomially distributed with a different rate for each database and that rates for each database were normally distributed. The first model was without covariates and with an uninformative prior for between database variability; the second model included covariates—for genotype, prior treatment and cirrhosis when starting the current treatment—so that data from each database could be considered exchangeable [13]. The third and fourth models were a repeat of the first two but with a weakly informative prior for between database variability. Models with covariates provide estimates of the SVR rate in a group of reference patients (here HCV genotype 1 patients, previously treated but now with decompensated cirrhosis); the model can then be used to estimate SVR rates in other covariate subgroups (such as previously treated genotype 1 patients with stable or no cirrhosis).

With a small number of databases, our uninformative prior for between database variability—a uniform distribution on the standard deviation of the between database variability—could lead to ‘under-pooling’ of data [13]. Therefore, in the last two models, we used a weakly informative half-Cauchy prior with its scale parameter set at the prior standard deviation [13] assuming that the lowest and highest SVR rates between databases would be covered with 95% probability by a range of odds ratios from 1/4 to 4 relative to the average across all databases.

Adding covariates essentially accounts for what could be material differences in patient mix between databases. Weakly informative priors were used for this adjustment: distributions where ‘the percentiles of the prior distribution would be viewed as at least reasonable if not liberally inclusive by all those working in the research topic’ [14]. Weakly informative priors restrict covariate effects to a range of values that is clinically sensible, ruling out extreme values that no knowledgeable clinician would find plausible [15]. The effects of genotype 3 and of genotypes other than 1 or 3 were both assumed to be of ‘uncertain direction’ (prior odds ratio (OR) 1.0, 95% credible interval (CI) 0.25 to 4.0); no prior treatment was assumed to be ‘possibly beneficial’ (prior OR 1.5, 95% CI 0.38 to 6.0); and no or stable cirrhosis was assumed to be ‘probably beneficial’ (prior OR 2.0, 95% CI 0.5 to 8.0) [16, 17]. There is clinical interest in the value of including ribavirin in treatment combinations but limited information [18, 19]. Given adequate data were collected on the use of daclatasvir and sofosbuvir both with and without ribavirin, the fourth model was extended to include an additional covariate for ribavirin use, with its effect assumed to be of ‘uncertain direction’ (prior OR 1.0, 95% CI 0.25 to 4.0).

We report estimated SVR rates and their 95% CI from each model. Fitting a sequence of models allowed us to check that as models became more complex, estimates varied between models in a logical fashion. However the most complex fourth model seems the most appropriate because it better meets the assumption of exchangeability between databases and gives rise to greater pooling of data across databases. Exchangeability implies that each database is independent of the others and, after adjusting for covariates, contains identically distributed data [13]. Models were fit using R 3.1.0, R2WinBUGS 2.1–20 and WinBUGS 1.4.3.

## Results

### Patients

**Fig. 1:**
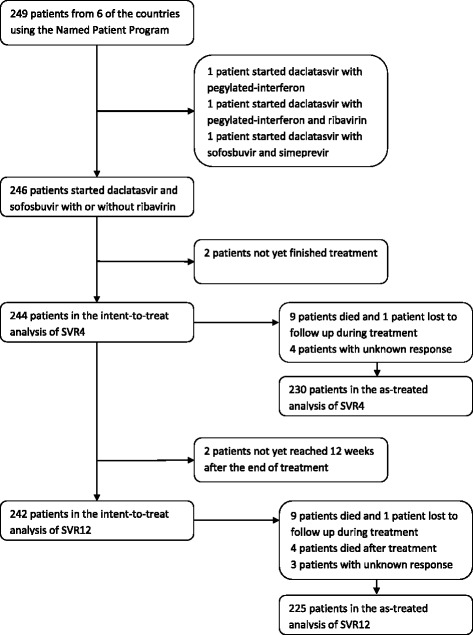
Patient flow. Patients included in intent-to-treat and as-treated analyses of sustained virologic response at four weeks after the end of treatment (SVR4) and at 12 weeks after the end of treatment (SVR12)

Of the 249 NPP patients identified in the six databases, 246 patients started daclatasvir and sofosbuvir (Fig. 1) with or without ribavirin (65 and 181 patients respectively). At the time of analysis, four of the 246 patients had not been followed for at least 12 weeks after the end of treatment, so that the SVR12 intent-to-treat analysis was of 242 patients. Of these 242 patients, 13 died prior to 12 weeks after treatment, one was lost to follow up during treatment and a response was missing for three patients so that the SVR12 as-treated analysis was of 225 patients. Of the 14 patients that died or were lost to follow up prior to 12 weeks after treatment, 13 had decompensated cirrhosis. The remaining patient received a second liver transplant during treatment and died before completing treatment.

Most of the 249 NPP patients were male (78%), with a median age of 56 years (Table 1). Most had HCV genotype 1 (75%), were treatment experienced (65%), had advanced fibrosis or cirrhosis (82% F3-F4) and decompensated cirrhosis (59%) and many had had a liver transplant (40%). Those given ribavirin were less likely to have decompensated cirrhosis (55%) and more likely to have had a liver transplant (44%) than those not given ribavirin (61% and 38% respectively). Liver transplantation during treatment was reported for 27 patients (11%); 21 of these patients had decompensated cirrhosis.

**Table 1:** Patient characteristics when starting hepatitis C (HCV) treatment with daclatasvir (*n =* 249)

Characteristics	All	Genotype			Ribavirin	
		1^a^	3^b^	Other^c^	With	Without
	*n =* 249	*n =* 187	*n =* 40	*n =* 22	*n =* 66	*n =* 183
Age, median (years)	56	56	55	55	55	57
Male (%)	78	75	85	91	85	76
HIV co-infection (%)	10	8	15	14	11	9
Prior HCV treatment (%)	65	69	48	64	65	65
Cirrhosis (%)						
- No	14	16	5	14	18	13
- Compensated	27	21	48	36	27	26
- Decompensated	59	63	48	50	55	61
Child Pugh class (%)						
- A	27	27	20	41	29	26
- B	33	37	23	14	33	32
- C	8	9	3	9	5	9
- Unknown	33	27	55	36	33	32
Fibrosis stage (%)						
- < F3	9	9	5	14	9	9
- F3 – F4	82	83	73	82	82	81
- Unknown	10	8	23	5	9	10
Hepatocellular carcinoma (%)						
- Never	58	56	65	68	61	57
- Past	17	19	13	14	18	17
- Current	12	13	10	9	15	12
- Unknown	12	12	13	9	6	14
Liver transplantation (%)						
- Never	34	31	50	32	27	36
- On list	13	14	13	9	11	14
- Before current treatment	40	40	33	50	44	38
- During current treatment	11	13	3	9	12	10
- Unknown	2	3	3	0	6	1

^a^Genotypes 1 (*n =* 14), 1a (*n =* 58), 1a/b (*n =* 3), and 1b (*n =* 112)

^b^Genotypes 3 (*n =* 18), 3a (*n =* 21) and 3 h (*n =* 1)

^c^Genotypes 2 (*n =* 4), 4 (*n =* 17) and 5 (*n =* 1)

For the 246 patients starting daclatasvir and sofosbuvir with or without ribavirin, the planned treatment duration was 24 weeks for all but three patients—these three patients were to receive 8, 12, and 20 weeks of treatment. Among the 243 patients that were to receive 24 weeks of treatment, the actual treatment duration was known for 218 patients: 187 (86%) were treated for between 23 and 25 weeks, 16 (7%) were treated for fewer than 23 weeks and 15 (7%) were treated for more than 25 weeks. Where known, the median duration of treatment was the same for those treated with and without ribavirin: 168 days (interquartile range 168 to 168) for those treated with ribavirin and 168 days (interquartile range 167 to 169) for those treated without ribavirin.

### Observed effectiveness

Of the 234 patients that completed treatment, an end of treatment response was missing for 15 patients; otherwise all patients had an end of treatment virologic response. 92% of intent-to-treat patients and 97% of as-treated patients had a SVR4; 90% of intent-to-treat patients and 97% of as-treated zpatients had a SVR12 (Table 2). Rates of SVR4 and SVR12 varied between countries, from 82 to 100%: this variability in response rates between countries was expected because in all countries except Spain, response rates were estimated from fewer than 30 patients.

**Table 2:** Observed sustained virological response rates at 4 weeks (SVR4) and at 12 weeks (SVR12) after completing therapy with daclatasvir and sofosbuvir, with or without ribavirin

Observed	SVR4				SVR12			
	As-treated		Intent-to-treat		As-treated		Intent-to-treat	
Country	%	Fraction	%	Fraction	%	Fraction	%	Fraction
Austria	100	14/14	100	14/14	100	13/13	93	13/14
Denmark	94	17/18	89	17/19	100	17/17	89	17/19
Spain	99	141/142	94	141/150	99	138/140	92	138/150
Sweden	100	15/15	94	15/16	100	15/15	94	15/16
Switzerland	85	23/27	82	23/28	85	22/26	85	22/26
UK	100	14/14	82	14/17	100	14/14	82	14/17
Overall	97	224/230	92	224/244	97	219/225	90	219/242

Of the six patients that failed to achieve a SVR12, all had decompensated cirrhosis, five were treatment experienced and none received ribavirin (Table 3). For one patient, the duration of treatment was not known and the end of treatment response was missing; the other five all received at least 20 weeks of treatment and had an end of treatment virologic response. Four of the five then had detectable viremia within 4 weeks after the end of treatment; the remaining patient had detectable viremia between 4 and 12 weeks after the end of treatment. Of the four patients with detectable viremia within 4 weeks: one was found to have Y93H resistance-associated variant (RAV) HCV; one had both Y93H and Q30H RAV HCV; the virus could not be sequenced for one patient but the patient was successfully re-treated with ledipasvir, sofosbuvir and ribavirin; the fourth patient was found to have L31M, L31I and Y93H RAV HCV but was also successfully re-treated with ledipasvir, sofosbuvir and ribavirin. The remaining patient with detectable viremia between 4 and 12 weeks was genotype 3a and was found to have Y93H RAV HCV.

**Table 3:** Observed sustained virological response rates in subgroups at 12 weeks (SVR12) after completing therapy with daclatasvir and sofosbuvir, with or without ribavirin

Subgroups		SVR12			
		As-treated		Intent-to-treat	
		%	Fraction	%	Fraction
Genotype^a^	1	98	171/174	92	171/186
	3	94	33/35	85	33/39
	Other	94	15/16	88	15/17
Cirrhosis	None or compensated	100	93/93	99	93/94
	Decompensated	95	126/132	85	126/148
Prior treatment	Naive	99	75/76	88	75/85
	Experienced	99	144/149	92	144/157
Ribavirin	Without	96	157/163	88	157/178
	With	100	62/62	97	62/64
Overall		97	219/225	90	219/242

^a^Genotype for the 6 patients without a sustained virologic response 12 weeks after the end of treatment: 1a, 1b (*n =* 2), 3a (*n =* 2) and 5a

### Estimated effectiveness

Estimates from the fourth hierarchical Bayesian model are shown for patients with HCV genotype 1, previously treated and now with decompensated cirrhosis (Table 4). These patients were selected as the reference group because they were typical of those treated in the NPP. For such patients, the estimated rate of SVR4 was 89% (95% CI 76 to 96%) and 96% (95% CI 86 to 100%) in intent-to-treat and as-treated analyses respectively; the estimated rate of SVR12 was 87% (95% CI 75 to 94%) and 97% (95% CI 89 to 100%) in intent-to-treat and as-treated analyses respectively. Estimates from all four models are shown in Additional file 1: Tables S1 to S4.

**Table 4:** Estimates from a hierarchical Bayesian model of the sustained virological response rates at 4 weeks (SVR4) and at 12 weeks (SVR12) after completing therapy with daclatasvir and sofosbuvir, with or without ribavirin

Estimate	SVR4				SVR12			
	As-treated		Intent-to-treat		As-treated		Intent-to-treat	
Country	%	95% CI	%	95% CI	%	95% CI	%	95% CI
Austria	97	87–100	92	81–99	98	89–100	88	75–96
Denmark	93	77–100	88	73–97	98	89–100	87	73–95
Spain	98	95–100	92	95–100	98	94–100	89	83–94
Sweden	96	79–100	90	73–100	97	85–100	87	72–96
Switzerland	82	56–97	82	59–94	82	57–97	84	64–93
UK	96	80–100	82	52–95	97	83–100	82	56–93
Overall	96	86–100	89	76–96	97	89–100	87	75–94

These estimates apply to patients with genotype 1 hepatitis C, previously treated and now with decompensated cirrhosis

The model provides estimates of the association between virologic response and covariates in the form of posterior OR relative to the response in reference patients. In the SVR12 intent-to-treat analysis, these estimates were: genotype 3, OR 0.69 (95% CI 0.25 to 1.54); other genotypes OR 1.06 (95% CI 0.32 to 2.77); no or stable cirrhosis, OR 6.73 (95% CI 2.42 to 16.1); no prior treatment, OR 0.92 (95% CI 0.39 to 1.89). Extending this model so that it includes an additional covariate for ribavirin use gives a posterior OR for this covariate of 2.36 (95% CI 0.86 to 5.42).

The model can then be used to estimate effectiveness in other covariate subgroups rather than in reference patients. Our results suggest a higher rate of SVR in NPP patients without decompensated cirrhosis. For patients with HCV genotype 1, previously treated but with stable or no cirrhosis, the estimated rate of SVR12 was 97% (95% CI 93 to 99%) and 99% (95% CI 96 to 100%) in intent-to-treat and as-treated analyses respectively. Our results also suggest that treatment might be more effective in NPP patients if it includes ribavirin. For patients with HCV genotype 1, previously treated and now with decompensated cirrhosis, the estimated rate of SVR12 in an intent-to-treat analysis was 92% (95% CI 81 to 98%) and 85% (95% CI 73 to 93%) with and without ribavirin respectively.

## Discussion

This study shows that 24 weeks of daclatasvir and sofosbuvir is an effective treatment for HCV in patients with advanced liver disease, with estimated rates of SVR12 of 87% (95% CI 75 to 94%) and 97% (95% CI 89 to 100%) in intent-to-treat and as-treated analyses respectively. The data are consistent with our expectation that it is easier to treat patients with no or stable cirrhosis because our posterior OR (6.73, 95% CI 2.42 to 16.1) suggests that treatment at an early stage of infection is even more beneficial than we anticipated (prior OR 2.0, 95% CI 0.5 to 8.0). The data do not support our expectation that it is easier to treat naive patients, because our posterior OR (0.92, 95% CI 0.39 to 1.89) suggests that treatment naivety is less beneficial than we anticipated (prior OR 1.5, 95% CI 0.38 to 6.0). Unfortunately there is little information in these data on how the rate of SVR varies with genotype: posterior ORs for genotype 3 and for other genotypes relative to genotype 1 (OR 0.69, 95% CI 0.25 to 1.54, and OR 1.06, 95% CI 0.32 to 2.77, respectively) are not materially different from our prior ORs (both 1.0, 95% CI 0.25 to 4.0). However since the intervals of these posterior ORs are contained within the interval of the prior OR, the data are consistent with our expectation that the rate of SVR does not vary markedly with genotype although the posterior OR for genotype 3 suggests this genotype could be harder to treat. Had we used an alternative prior for genotype 3 reflecting an expectation that genotype 3 would be harder to treat (prior OR 0.67, 95% CI 0.17 to 2.7), we would have found weak support in the data for this position (posterior OR 0.52, 95% CI 0.20 to 1.14)

In an additional analysis, we extended our model to include a covariate for ribavirin use. The shift in OR for this covariate (prior OR 1.0, 95% CI 0.25 to 4.0; posterior OR 2.36, 95% CI 0.86 to 5.42), suggests ribavirin use might be beneficial in these patients. Patients that received ribavirin do not seem materially different from those that did not (Table 1) and both groups were treated for a similar duration. The estimated rate of SVR12 in an intent-to-treat analysis was 92% (95% CI 81 to 98%) and 85% (95% CI 73 to 93%) with and without ribavirin respectively. Other studies suggest that adding ribavirin to this combination is beneficial in patients with decompensated cirrhosis [20, 21] but unnecessary when treating patients without cirrhosis [8]; a pattern seen in other oral interferon-free treatment combinations [19].

The strengths of this study include data collected in clinical practice and from patients with advanced liver disease including many patients with decompensated cirrhosis (59%) that previously were difficult to treat. Our data come from a number of European countries and estimates have been appropriately averaged over different health care systems. The limitations include a higher level of missing supporting data than would be expected in a controlled trial. Most patients in this study were infected with HCV genotype 1, so that we cannot reliably estimate the effectiveness of this treatment combination in other genotypes or draw conclusions about whether effectiveness differs between genotypes. And most countries contributed only a small number of patients, so that we needed to use advanced statistical methods to sensibly combine data but then more effort is required to interpret our results.

The results of this study are consistent with results from recently completed Phase 2 and 3 studies and with recently published or preliminary results from other early access programmes. In recent Phase 2 and 3 studies, the treatment combination of daclatasvir and sofosbuvir achieved a rate of SVR12 above 95% in genotype 1 patients without cirrhosis [8]; rates above 95% in both naive and previously treated genotype 1 patients co-infected with HIV when treated for 12 weeks [22], and a rate above 95% in genotype 3 patients without cirrhosis [23]. A number of Phase 3 studies of daclatasvir and sofosbuvir (with or without ribavirin) have included at least some patients with cirrhosis and results suggest that lower rates of SVR12 are to be expected: 63% of 32 genotype 3 patients with compensated cirrhosis achieved a SVR12 after 12 weeks of treatment (without ribavirin) [23]; 86% of 36 genotype 3 patients with compensated cirrhosis achieved a SVR12 after 12 or 16 weeks of treatment (with ribavirin) [21]; and 79% of 34 genotype 1 patients with decompensated cirrhosis (Child-Pugh class B or C) achieved a SVR12 after 12 weeks of treatment (with ribavirin) [24]. Data from other early access programmes suggest that this treatment combination is just as effective after liver transplantation [25, 26] but effectiveness is reduced in patients with HCV genotype 3 or decompensated cirrhosis [20, 26, 27].

## Conclusions

Patients with decompensated cirrhosis have always been difficult to treat [1, 28]. With direct acting antivirals, efficacy is reduced in such patients and it is not yet clear which treatments are best [28–30]. For the moment, the recommended treatment is sofosbuvir in combination with either daclatasvir, ledipasvir or velpatasvir [11, 12] but such recommendations are frequently updated. This study provides evidence that the combination of daclatasvir and sofosbuvir is an effective treatment in clinical practice for HCV genotype 1 patients with decompensated cirrhosis. The study adds to limited evidence that, for these patients, treatment is more effective if ribavirin is added to the combination [20, 21]. Other questions remain: whether fewer than 24 weeks of treatment erodes effectiveness in these patients – and by how much [20, 21, 23, 24, 27]; whether this treatment is as effective in HCV genotype 3 patients with decompensated cirrhosis [20, 21, 23, 26]; and whether the combination of daclatasvir and sofosbuvir is more effective than other treatment combinations for patients with decompensated cirrhosis [20].

## Additional file

Additional file 1: Tables S1 to S4. This file consists of a title page, a table of contents, a brief introduction to hierarchical Bayesian modelling, references and four tables of results (one table per page). (DOC 90 kb)

## Abbreviations

95% CI: 95% credible interval
BMS: Bristol-Myers Squibb
HCV: Hepatitis C virus
NPP: Named Patient Programme
OR: Odds ratio
SVR: Sustained virologic response
SVR12: Sustained virologic response at 12 weeks after the end of treatment
SVR4: Sustained virologic response at four weeks after the end of treatment
