# Supplementary material for: The effectiveness of daclatasvir based therapy in European patients with chronic hepatitis C and advanced liver disease
Source: BMC Infect Dis. 2017 Jan 7;17:45. doi: 10.1186/s12879-016-2106-x (PMC5219681; doi:10.1186/s12879-016-2106-x)
Supplement: Additional file 1: Tables S1 to S4. — This file consists of a title page, a table of contents, a brief introduction to hierarchical Bayesian modelling, references and four tables of results (one table per page). (DOC 90 kb) [file 12879_2016_2106_MOESM1_ESM.doc]

# The effectiveness of daclatasvir based therapy in European patients with chronic hepatitis C and advanced liver disease

Jim Young^1*^, Nina Weis^2^, Harald Hofer^3^, William Irving^4^, Ola Weiland^5^, Emiliano Giostra^6^,
Juan Manuel Pascasio^7^, Lluis Castells^8^, Martin Prieto^9^, Roelien Postema^10^, Cinira Lefevre^11^,
 David Evans^11^, Heiner C. Bucher^1#^, Jose Luis Calleja^12^

^1^ Basel Institute for Clinical Epidemiology and Biostatistics, University Hospital Basel, Basel, Switzerland

^2^ Department of Infectious Diseases, Copenhagen University Hospital, Hvidovre, Denmark

^3^ Department of Internal Medicine III, Division of Gastroenterology and Hepatology, Medical University of Vienna, Vienna, Austria

^4^ NIHR Nottingham Digestive Diseases Biomedical Research Unit, University of Nottingham, UK

^5^ Division of Infectious Diseases, Department of Medicine, Karolinska Institutet, Karolinska University Hospital Huddinge, Stockholm, Sweden

^6^ Service de Gastroentérologie et Hépatologie, Hôpitaux universitaires de Genève, Geneva, Switzerland

^7^ Hospital Universitario Virgen del Rocío, Seville, Spain

^8^ Liver Unit, Internal Medicine Department, Hospital Universitari Vall Hebron, Barcelona, Spain

^9^ Hepatology Unit, Hospital Universitario y Politécnico La Fe, Valencia, Spain

^10^ Worldwide Health Economics and Outcomes Research, Bristol-Myers Squibb, Uxbridge, United Kingdom

^11^ Worldwide Health Economics and Outcomes Research, Bristol-Myers Squibb, Rueil-Malmaison, France

^12^ Liver Unit, Hospital Universitario Puerta de Hierro, Universidad Autonoma de Madrid, Madrid, Spain.

^*^ Corresponding author
Basel Institute for Clinical Epidemiology and Biostatistics, University Hospital Basel, Spitalstrasse 12, CH-4031 Basel, Switzerland. Tel. +41 61 556 5100; fax: +41 61 265 3109.
Email address: james.young@usb.ch

# Supplementary Material – Table of Contents

Hierarchical Bayesian modelling page 3

References page 4

Table S1: Estimates from the first hierarchical Bayesian model page 5

Table S2: Estimates from the second hierarchical Bayesian model page 6

Table S3: Estimates from the third hierarchical Bayesian model page 7

Table S4: Estimates from the fourth hierarchical Bayesian model page 8

# Hierarchical Bayesian modelling

We used hierarchical Bayesian modelling for this analysis rather than logistic regression. The latter requires large samples – small samples introduce a bias away from the null leading to overestimates of model parameters [1]. In the context of logistic regression, sample size is assessed by the number of events (successes or failures, whichever is less frequent) with a rule of thumb that 10 events are needed per model parameter [2]. Our data has too few events to support logistic regression models that would allow for differences in response rates between databases. For example, there were only six failures in the as-treated analysis of sustained virological response at 12 weeks after completing therapy (Table 2 of the published article).

We estimated virologic response rates using four hierarchical Bayesian models of increasing complexity. In all models we assumed responses were binomially distributed with a different rate for each database and that response rates for each database were normally distributed. The first model was without covariates and with an uninformative prior for between database variability. The second model included covariates – for genotype, prior treatment and cirrhosis when starting the current treatment. The third and fourth models were a repeat of the first two but with a weakly informative prior for between database variability. Here we show estimates for all four models (Tables S1 to S4). Estimates for the fourth model are shown in Table 4 of the published article but are shown here as well so that estimates can be compared between models.

Models without covariates provide estimates of a response rate averaged across all patients. Models with covariates provide estimates of the response rate in a group of reference patients (here HCV genotype 1 patients, previously treated but now with decompensated cirrhosis). For this reason, estimates from Models 1 and 3 tend to be higher than those from Models 2 and 4, because averaging across all patients includes patients with no or stable cirrhosis and such patients are easier to treat than patients with decompensated cirrhosis. In Models 2 and 4, we specifically estimate response rates for those harder to treat patients with decompensated cirrhosis.

When a weakly informative prior is used to estimate database variability (Models 3 and 4), estimates for response rates within each database tend to be closer to the overall estimate than if an uninformative prior is used (Models 1 and 2). This is most apparent in the estimated response rates for Switzerland. This is because with few databases, there is little information in the data about the variability between databases. With an uninformative prior, the prior is likely to be unrealistically broad and this results in an overestimate of database variability and less pooling of data than a weakly informative prior which limits the estimate of database variability to a narrower and more realistic interval [3].

# References

1. Nemes S, Jonasson JM, Genell A, Steineck G. Bias in odds ratios by logistic regression modelling and sample size. BMC Med Res Methodol 2009; 9:56.

2. Peduzzi P, Concato J, Kemper E, Holford TR, Feinstein AR. A simulation study of the number of events per variable in logistic regression analysis. J Clin Epidemiol 1996; 49:1373-9.

3. Gelman A, Carlin JB, Stern HS, Dunson DB, Vehtari A, and Rubin DB. Bayesian data analysis. Third edition. Boca Raton: CRC Press, 2013. Chapters 5.2 Exchangeability and setting up hierarchical models, 5.7 Weakly informative priors for hierarchical variance parameters.

**Table S1** Estimates from the first hierarchical Bayesian model of the sustained virological response rates at 4 weeks (SVR4) and at 12 weeks (SVR12) after completing therapy with daclatasvir and sofosbuvir, with or without ribavirin. These estimates apply to all patients.

| Estimate | SVR4 | | | | SVR12 | | | |
| --- | --- | --- | --- | --- | --- | --- | --- | --- |
|  | As-treated | | Intent-to-treat | | As-treated | | Intent-to-treat | |
| Country | % | 95% CI | % | 95% CI | % | 95% CI | % | 95% CI |
| Austria | 99 | 93 - 100 | 94 | 85 - 100 | 100 | 95 - 100 | 90 | 80 - 97 |
| Denmark | 95 | 83 - 100 | 90 | 78 - 97 | 100 | 96 - 100 | 90 | 80 - 96 |
| Spain | 99 | 97 - 100 | 93 | 89 - 97 | 99 | 96 - 100 | 91 | 87 - 95 |
| Sweden | 99 | 93 - 100 | 92 | 81 - 99 | 100 | 96 - 100 | 91 | 82- 97 |
| Switzerland | 87 | 72 - 97 | 87 | 72 - 95 | 85 | 69 - 96 | 88 | 77 - 95 |
| UK | 99 | 93 - 100 | 88 | 71 - 96 | 100 | 96 - 100 | 88 | 74 - 95 |
| Overall | 97 | 80 - 100 | 91 | 81 - 98 | 97 | 67 -100 | 90 | 82 - 95 |

**Table S2** Estimates from the second hierarchical Bayesian model of the sustained virological response rates at 4 weeks (SVR4) and at 12 weeks (SVR12) after completing therapy with daclatasvir and sofosbuvir, with or without ribavirin. These estimates apply to patients with genotype 1 hepatitis C, previously treated and now with decompensated cirrhosis.

| Estimate | SVR4 | | | | SVR12 | | | |
| --- | --- | --- | --- | --- | --- | --- | --- | --- |
|  | As-treated | | Intent-to-treat | | As-treated | | Intent-to-treat | |
| Country | % | 95% CI | % | 95% CI | % | 95% CI | % | 95% CI |
| Austria | 98 | 87 - 100 | 94 | 81 - 100 | 99 | 94 - 100 | 88 | 74 - 98 |
| Denmark | 93 | 76 - 100 | 88 | 70 - 98 | 99 | 95 - 100 | 87 | 70 - 96 |
| Spain | 99 | 89 - 100 | 93 | 87 - 97 | 98 | 95 - 100 | 89 | 83 - 95 |
| Sweden | 97 | 83 - 100 | 90 | 69 - 99 | 99 | 91 - 100 | 87 | 70 - 98 |
| Switzerland | 77 | 51 - 95 | 78 | 55 - 93 | 77 | 52 - 94 | 82 | 60 - 93 |
| UK | 97 | 81 - 100 | 77 | 43 - 95 | 99 | 90 - 100 | 79 | 48 - 94 |
| Overall | 95 | 66 - 100 | 88 | 66 - 98 | 97 | 63 - 100 | 86 | 71 - 95 |

**Table S3** Estimates from the third hierarchical Bayesian model of the sustained virological response rates at 4 weeks (SVR4) and at 12 weeks (SVR12) after completing therapy with daclatasvir and sofosbuvir, with or without ribavirin. These estimates apply to all patients.

| Estimate | SVR4 | | | | SVR12 | | | |
| --- | --- | --- | --- | --- | --- | --- | --- | --- |
|  | As-treated | | Intent-to-treat | | As-treated | | Intent-to-treat | |
| Country | % | 95% CI | % | 95% CI | % | 95% CI | % | 95% CI |
| Austria | 98 | 92 - 100 | 93 | 85 - 99 | 98 | 92 - 100 | 90 | 82 - 96 |
| Denmark | 96 | 86 - 100 | 91 | 80 - 97 | 98 | 93 - 100 | 90 | 81 - 96 |
| Spain | 99 | 97 - 100 | 93 | 89 - 96 | 98 | 96 – 100 | 91 | 87 - 95 |
| Sweden | 98 | 92 - 100 | 92 | 82 - 98 | 98 | 93 – 100 | 90 | 83 - 96 |
| Switzerland | 90 | 75 - 98 | 88 | 75 - 95 | 90 | 74 – 98 | 89 | 79 - 94 |
| UK | 98 | 92 - 100 | 89 | 75 - 95 | 98 | 92 – 100 | 89 | 77 - 95 |
| Overall | 98 | 93 - 100 | 91 | 84 - 97 | 98 | 94 - 100 | 90 | 84 - 94 |

**Table S4** Estimates from the fourth hierarchical Bayesian model of the sustained virological response rates at 4 weeks (SVR4) and at 12 weeks (SVR12) after completing therapy with daclatasvir and sofosbuvir, with or without ribavirin. These estimates apply to patients with genotype 1 hepatitis C, previously treated and now with decompensated cirrhosis.

| Estimate | SVR4 | | | | SVR12 | | | |
| --- | --- | --- | --- | --- | --- | --- | --- | --- |
|  | As-treated | | Intent-to-treat | | As-treated | | Intent-to-treat | |
| Country | % | 95% CI | % | 95% CI | % | 95% CI | % | 95% CI |
| Austria | 97 | 87 - 100 | 92 | 81 - 99 | 98 | 89 - 100 | 88 | 75 - 96 |
| Denmark | 93 | 77 - 100 | 88 | 73 - 97 | 98 | 89 - 100 | 87 | 73 - 95 |
| Spain | 98 | 95 - 100 | 92 | 95 - 100 | 98 | 94 - 100 | 89 | 83 - 94 |
| Sweden | 96 | 79 - 100 | 90 | 73 - 100 | 97 | 85 - 100 | 87 | 72 - 96 |
| Switzerland | 82 | 56 - 97 | 82 | 59 - 94 | 82 | 57 - 97 | 84 | 64 - 93 |
| UK | 96 | 80 - 100 | 82 | 52 - 95 | 97 | 83 - 100 | 82 | 56 - 93 |
| Overall | 96 | 86 - 100 | 89 | 76 - 96 | 97 | 89 - 100 | 87 | 75 - 94 |
